# Prognostic value of sarcopenia in patients with lung cancer treated with epidermal growth factor receptor tyrosine kinase inhibitors or immune checkpoint inhibitors

**DOI:** 10.3389/fnut.2023.1113875

**Published:** 2023-03-08

**Authors:** Jiahua Lyu, Ningjing Yang, Ling Xiao, Xinyu Nie, Jing Xiong, Yudi Liu, Min Zhang, Hangyue Zhang, Cunhan Tang, Shiyi Pan, Long Liang, Hansong Bai, Churong Li, Hao Kuang, Tao Li

**Affiliations:** ^1^School of Medicine, University of Electronic Science and Technology of China, Chengdu, China; ^2^Sichuan Cancer Hospital, School of Medicine, University of Electronic Science and Technology of China, Chengdu, China

**Keywords:** sarcopenia, lung cancer, immune-checkpoint inhibitors, EGFR-TKIs, prognosis

## Abstract

**Objectives:**

It remains controversial whether sarcopenia has any significant impact on the efficacy of epidermal growth factor receptor tyrosine kinase inhibitors (EGFR-TKIs) or immune checkpoint inhibitors (ICIs) in patients with advanced non-small cell lung cancer (NSCLC). Therefore, in this study, we aimed to assess the association between sarcopenia and clinical outcomes in patients with advanced NSCLC receiving EGFR-TKIs or ICIs as a first-line therapy.

**Methods:**

We retrospectively enrolled 131 patients with advanced NSCLC treated with first-line EGFR-TKIs or ICIs between 1 March 2019 and 31 March 2021. To estimate sarcopenia, we calculated skeletal muscle index (SMI) as the ratio of skeletal muscle area (cm^2^) to height squared (m^2^). Associations between sarcopenia and overall survival (OS) and progression-free survival (PFS) were evaluated using the Kaplan–Meier method and log-rank tests, respectively. A Cox proportional hazards regression model was used to assess the factors associated with OS and PFS. The Student’s *t*-test or Mann–Whitney U test was used to compare the SMI between patients with or without objective response and disease control. The chi-squared test was used to compare adverse events (AEs) between patients with and without sarcopenia.

**Results:**

Among the 131 patients, 35 (26.7%) were diagnosed with sarcopenia. Sarcopenia was an independent predictor of poor OS and PFS (*p* < 0.05) overall and in the EGFR-TKI- and ICI-treated cohorts. Among all patients, those with sarcopenia showed significantly shorter OS and PFS than those without sarcopenia (median OS and PFS: 13.0 vs. 26.0 months and 6.4 vs. 15.1 months; both *p* < 0.001). These associations were consistent across the subtypes of most clinical characteristics. Statistically significant differences between the objective response (OR) and non-OR groups were also observed in the mean SMI (OR group, 43.89 ± 7.55 vs. non-OR group, 38.84 ± 7.11 cm^2^/m^2^; *p* < 0.001). In addition, we observed similar results with disease control (DC) and non-DC groups (DC group, 42.46 ± 7.64 vs. non-DCR group, 33.74 ± 4.31 cm^2^/m^2^; *p* < 0.001). The AEs did not differ significantly between the sarcopenia and non-sarcopenia groups.

**Conclusion:**

Sarcopenia before treatment might be a significant predictor of poor clinical outcomes (shorter OS and PFS, fewer ORs, less DC) in patients with advanced NSCLC treated with EGFR-TKIs or ICIs as the first-line therapy.

## 1. Introduction

Lung cancer is the second most common malignant tumor and the leading cause of cancer-related deaths worldwide. Non-small cell lung cancer (NSCLC) comprises the majority (85%) of all lung cancers (1). Approximately, 25% of NSCLC patients present with an advanced stage at initial diagnosis (2), with a 5-year survival rate of less than 20% (3).

Epidermal growth factor receptor-tyrosine kinase inhibitors (EGFR-TKIs) and immune checkpoint inhibitors (ICIs) have been shown to significantly improve the survival of metastatic NSCLC patients with mutant and wild-type EGFR, respectively (4, 5). However, not all eligible patients can benefit equally from EGFR-TKIs or ICIs (6, 7). Although EGFR mutation and the PD-L1 expression level have been reported as potential predictors of the therapeutic efficacy for EGFR-TKIs and ICIs, it is essential to identify additional biomarkers that can help determine those patients most likely to benefit from these therapies.

Sarcopenia is characterized by progressive loss of skeletal muscle strength and mass and is associated with decreased muscle protein synthesis and increased protein degradation (8, 9). Sarcopenia has been reported in approximately 50% of lung cancer patients and is associated with a decrease in the efficacy of surgery or chemotherapy, toxicity, and a worse quality of life (10, 11). However, the impact of sarcopenia on the efficacy and toxicity of EGFR-TKIs and ICIs remains unclear.

Several articles have suggested that sarcopenia is correlated with poor clinical outcomes in patients receiving PD-1/PD-L1 inhibitors (12, 13), however, other researches have reached inconsistent conclusions (14–16). The same controversy also exists In NSCLC patients treated with EGFR-TKIs. A retrospective study showed that sarcopenia did not affect the response to gefitinib in patients with EGFR-mutated NSCLC (17). In contrast, another retrospective study enrolling 72 NSCLC patients treated with erlotinib found that sarcopenia was a negative biomarker that was significantly associated with response and survival outcomes (18).

In brief, there is no consensus as to whether sarcopenia is a prognostic biomarker for EGFR-TKI or ICI treatment of advanced NSCLC, especially when used as the first-line treatment. Consequently, we sought to investigate the potential predictive value of sarcopenia on the efficacy of EGFR-TKIs in NSCLC patients harboring EGFR mutations or of ICIs in patients with wild-type EGFR.

## 2. Materials and methods

### 2.1. Patients

In this study, we retrospectively collected the data of patients with pathologically confirmed metastatic NSCLC who were treated with EGFR-TKIs or ICIs as the first-line therapy at Sichuan Cancer Hospital, China, from 16 January 2018 to 8 June 2021. Patients who met the inclusion criteria below were enrolled: (1) histologically confirmed stage IV metastatic NSCLC; (2) treated with EGFR-TKIs or ICIs as first-line therapies; and (3) underwent a chest/abdominal CT scan within 4 weeks prior to EGFR-TKI or ICI therapy. This study was approved by the Ethics Committee of Sichuan Cancer Hospital and carried out in strict accordance with the Declaration of Helsinki.

### 2.2. Data collection

We consecutively enrolled 205 patients with advanced NSCLC treated with EGFR-TKIs or ICIs in our hospital. After selection according to the inclusion and exclusion criteria, 54 eligible patients receiving ICIs and 77 eligible patients receiving EGFR-TKIs were included (Supplementary Figure 1).

Subsequently, we obtained basic demographic and clinical data for all eligible patients, including age, sex, history of smoking and alcohol consumption, Karnofsky performance status (KPS) score, histopathology, height, weight, routine biochemical and hematological test results, CT images, EGFR mutation status, PD-L1 expression before EGFR-TKI or ICI initiation, treatment option, treatment response, and toxicity. The follow-up date ended at the date of the outcome event, the date of death, or the end of follow-up, whichever came first.

### 2.3. Skeletal muscle measurement and definition of sarcopenia

The chest/abdomen CT scan for sarcopenia evaluation was obtained within 4 weeks before the start of EGFR-TKIs or ICIs. The skeletal muscle area was measured at the L3 level by two experienced radiologists (NJY and XYN) using sliceOmatic (TomoVision 5.0, Magog, QC, Canada) with –29 to 150 Hounsfield unit (HU) (Supplementary Figure 2). The skeletal muscle index (SMI) was calculated using the formula (L3 muscle area in cm^2^)/(patient height in m^2^). Sarcopenia was defined as a low SMI as follows: (1) for women, SMI < 31.6 cm^2^/m^2^; (2) for men, SMI < 40.2 cm^2^/m^2^ (19).

### 2.4. Follow-up

Tumor response evaluations were performed based on chest CT according to the Response Evaluation Criteria in Solid Tumours (RECIST) version 1.1 (for patients receiving EGFR-TKIs) or iRECIST criteria (for patients receiving ICIs). PFS and overall survival (OS) were calculated from the date of initiation of EGFR-TKI or ICI treatment to the date of progression (for PFS) or patient death (for OS) or to the last follow-up. The incidence and severity of all adverse events (AEs) were monitored and evaluated according to the National Cancer Institute Common Terminology Criteria for Adverse Events (NCI-CTCAE V5.0).

### 2.5. Statistical analysis

We used R software, version 4.0.2 (R Foundation for Statistical Computing), for statistical analysis. Multiple Cox regression analyses were conducted to evaluate the impact of sarcopenia and other candidate prognostic factors on the OS and PFS. The Kaplan–Meier method and log-rank test were used to compare OS and PFS between patients with and without sarcopenia. The association between the presence of sarcopenia and demographic, clinical, and laboratory parameters, treatment response, and occurrence of AEs was established using the exact Fisher test and χ^2^ test. Scatter plots were used to graphically represent the association between sarcopenia and hemoglobin, total protein, albumin, prealbumin, serum triglyceride, and serum cholesterol levels and BMI using Spearman’s correlation. All statistics were two-tailed, and *p*-values ≤ 0.05 were considered statistically significant.

## 3. Results

### 3.1. Patient characteristics

The baseline characteristics of the 131 enrolled patients are presented in Table 1. A total of 77 and 54 patients received first-line treatment with EGFR-TKIs or ICIs, respectively. Patients were categorized into two groups (sarcopenia and non-sarcopenia) according to the previously defined criteria for sarcopenia. Among all patients, 35 (26.7%) were diagnosed with sarcopenia, including 17 EGFR-TKI-treated and 18 ICI-treated patients. A full comparison of the baseline characteristics between the sarcopenia and non-sarcopenia groups is presented in Table 1. Sarcopenia was significantly more common in patients who had smoked (*p* = 0.038) or drunk (*p* = 0.041) regularly, or those who had a low KPS score (*p* = 0.002) or low albumin (*p* = 0.023). The baseline characteristics of the EGFR-TKI and ICI cohorts are listed in the Supplementary Tables 1, 2, respectively.

**TABLE 1:** Baseline characteristics of all patients.

Characteristic	Non-sarcopenia	Sarcopenia	*p*-value
*N*	96(73.3%)	35(26.7%)	
Sex, *n* (%)			0.076
Female	44 (45.8%)	10 (28.6%)	
Male	52 (54.2%)	25 (71.4%)	
Smoking, *n* (%)			**0.038**
No	58 (60.4%)	14 (40%)	
Yes	38 (39.6%)	21 (60%)	
Drinking, *n* (%)			**0.041**
No	77 (80.2%)	22 (62.9%)	
Yes	19 (19.8%)	13 (37.1%)	
Histopathology, *n* (%)			0.764
AC	79 (82.3%)	28 (80%)	
SCC	17 (17.7%)	7 (20%)	
EGFR mutations, *n* (%)			0.152
No	36 (37.5%)	18 (51.4%)	
Yes	60 (62.5%)	17 (48.6%)	
EGFR mutation sites, *n* (%)			0.142
None	36 (37.5%)	18 (51.4%)	
Exon 19	34 (35.4%)	13 (37.1%)	
Exon 20	1 (1%)	1 (2.9%)	
Exon 21	25 (26%)	3 (8.6%)	
PD-L1 expression, *n* (%)			0.081
<1%	15 (15.6%)	7 (20%)	
1–50%	12 (12.5%)	10 (28.6%)	
>50%	10 (10.4%)	1 (2.9%)	
Unknown	59 (61.5%)	17 (48.6%)	
Chemotherapy, *n* (%)			0.237
No	52 (54.2%)	23 (65.7%)	
Yes	44 (45.8%)	12 (34.3%)	
EGFR-TKI therapy, *n* (%)			0.152
No	36 (37.5%)	18 (51.4%)	
Yes	60 (62.5%)	17 (48.6%)	
EGFR-TKI drugs, *n* (%)			0.176
None	36 (37.5%)	18 (51.4%)	
1st generation	26 (27.1%)	5 (14.3%)	
2nd generation	5 (5.2%)	0 (0%)	
3rd generation	29 (30.2%)	12 (34.3%)	
ICI therapy, *n* (%)			0.152
No	60 (62.5%)	17 (48.6%)	
Yes	36 (37.5%)	18 (51.4%)	
ICI drugs, *n* (%)			0.053
None	60 (62.5%)	17 (48.6%)	
PD-1 PD-L1	35 (36.5%) 1 (1%)	15 (42.9%) 3 (8.6%)	
KPS score, *n* (%)			**0.002**
70	4 (4.2%)	6 (17.1%)	
80	49 (51%)	24 (68.6%)	
90	42 (43.8%)	4 (11.4%)	
100	1 (1%)	1 (2.9%)	
Age (years), mean ± SD	59.08 ± 9.93	59.09 ± 10.20	0.999
BMI, mean ± SD	22.91 ± 3.03	22.12 ± 2.97	0.188
Hemoglobin, mean ± SD	126.61 ± 18.04	122.71 ± 16.14	0.263
hCRP, median (IQR)	3.49 (0.77, 11.55)	6.77 (1.69, 22.07)	0.165
Total protein, mean ± SD	65.45 ± 6.31	64.06 ± 4.72	0.239
Albumin, mean ± SD	38.64 ± 4.85	36.54 ± 3.95	**0.023**

Bold values mean *p* < 0.05. SCC, squamous cell carcinoma; AC, adenocarcinoma; EGFR-TKI, epidermal growth factor receptor tyrosine kinase inhibitors; ICI, immune checkpoint inhibitor; PD-1, programmed death-1; PD-L1, programmed cell death-ligand 1; KPS, Karnofsky performance status; SD, standard deviation; BMI, body mass index; IQR, interquartile range; hCRP, hypersensitive C-reactive protein.

**FIGURE 1:**
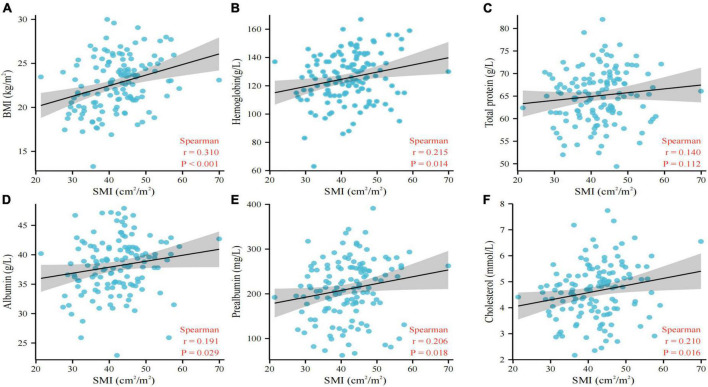
Association between BMI **(A)**, hemoglobin **(B)**, total protein **(C)**, albumin **(D)**, prealbumin **(E)**, cholesterol **(F)**, and L3 SMI. BMI, body mass index; SMI, skeletal muscle index.

The relationship between nutritional status and L3 SMI is shown in Figure 1. The scatter plot shows a high correlation between nutritional status factors, including BMI (*p* < 0.001, Figure 1A), hemoglobin level (*p* = 0.014, Figure 1B), albumin level (*p* = 0.029, Figure 1D), prealbumin level (*p* = 0.018, Figure 1E), and cholesterol level (*p* = 0.016, Figure 1F), and L3 SMI, indicating that poor nutritional status is a risk factor for sarcopenia in NSCLC patients.

### 3.2. Effect of sarcopenia on OS and PFS

**FIGURE 2:**
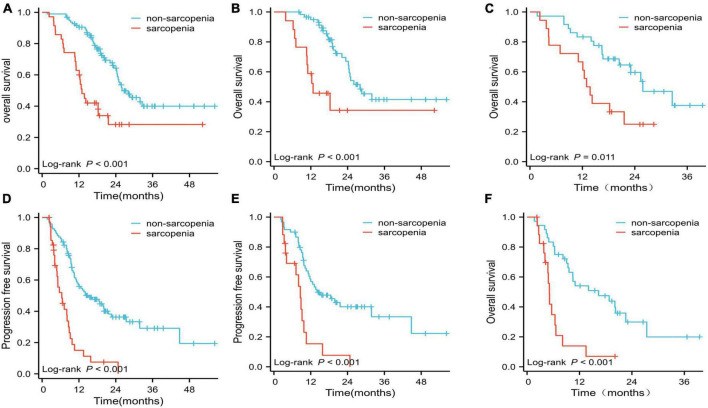
Overall survival (OS) and progression-free survival (PFS) curves. **(A)** OS for the two groups in the whole cohort; **(B)** OS for the two groups treated with EGFR-TKIs; **(C)** OS for the two groups treated with ICIs; **(D)** PFS for the sarcopenia and non-sarcopenia groups in the whole cohort; **(E)** PFS for the two groups treated with EGFR-TKIs; **(F)** PFS for the two groups treated with ICIs.

The Kaplan–Meier curves for OS and PFS grouped by sarcopenia and non-sarcopenia are shown in Figure 2. Analysis of the entire patient cohort showed a significant difference in OS between patients with sarcopenia and those without sarcopenia, with a median OS of 13 and 26 months, respectively (*p* < 0.001; Figure 2A). Kaplan–Meier analysis also revealed that patients with sarcopenia had a significantly shorter PFS than those without sarcopenia (6.4 months vs. 15.1 months, *p* < 0.001; Figure 2D).

In the EGFR-TKI-treated cohort (*n* = 77), the median OS and PFS for patients with sarcopenia were significantly shorter than those for patients without sarcopenia (OS: 12.7 vs. 28.0 months; PFS: 8.6 vs. 14.1 months, respectively; both *p* < 0.001; Figures 2B, E). The median OS and PFS for patients with sarcopenia in the ICI-treated cohort were significantly shorter than those for patients without sarcopenia (OS: 13.4 vs. 25.8 months, *p* = 0.011; PFS: 5.1 vs. 16.4 months, *p* < 0.001; Figures 2C, F).

In the entire patient cohort, univariate Cox regression analysis revealed that BMI, sarcopenia, KPS score, levels of hemoglobin, hypersensitive C-reactive protein (hCRP) and albumin were significant prognostic factors for OS (all *p* < 0.05; Table 2). Multivariate analysis of all the above potential factors identified only sarcopenia [hazard ratio (HR): 2.187, 95% confidence interval (CI): 1.230–3.891, *p* = 0.008; Table 2] and albumin [hazard ratio (HR): 0.921, 95% confidence interval (CI): 0.860–0.987, *p* = 0.019; Table 2] as strong independent predictors of OS. Univariate and multivariate Cox regression analyses of both the EGFR-TKI-treated and ICI-treated cohorts confirmed that sarcopenia was an independent negative factor for OS (EGFR-TKI-treated group, HR: 2.806, 95% CI: 1.304–6.037, *p* = 0.008; Supplementary Table 3; ICI-treated group, HR: 2.155, 95% CI: 1.107–4.484, *p* = 0.028; Supplementary Table 4).

**TABLE 2:** Results of Cox regression analysis for overall survival for all patients.

Characteristics	Total (n)	Univariate analysis		Multivariate analysis	
		**Hazard ratio (95% CI)**	***p*-value**	**Hazard ratio (95% CI)**	***p*-value**
Sex	131				
Female	54	Reference			
Male	77	1.210 (0.729–2.010)	0.460		
Age	131	1.005 (0.980–1.031)	0.689		
Smoking	131				
No	72	Reference			
Yes	59	1.119 (0.684–1.830)	0.655		
Drinking	131				
No	99	Reference			
Yes	32	1.205 (0.691–2.101)	0.510		
Histopathology	131				
AC	107	Reference			
SCC	24	1.258 (0.668–2.370)	0.477		
Ch‘emotherapy	131				
No	75	Reference			
Yes	56	1.070 (0.652–1.754)	0.789		
EGFR-TKIs therapy	131				
Yes	77	Reference			
No	54	1.389 (0.848–2.274)	0.192		
Body mass index	131	0.918 (0.844–1.000)	0.050	0.948 (0.866–1.038)	0.252
Sarcopenia status	131				
Non-sarcopenia	63	Reference		Reference	
Sarcopenia	68	2.940 (1.744–4.956)	**<0.001**	2.187 (1.230–3.891)	**0.008**
KPS score	131				
70	10	Reference		Reference	
80	73	0.423 (0.188–0.951)	**0.037**	0.437 (0.178–1.074)	0.071
90	46	0.180 (0.074–0.439)	**<0.001**	0.358 (0.128–1.001)	**0.050**
100	2	0.000 (0.000–Inf)	0.996	0.000 (0.000–Inf)	0.996
Hemoglobin	131	0.982 (0.969–0.995)	**0.005**	1.000 (0.983–1.018)	0.967
hCRP	131	1.010 (1.001–1.019)	**0.035**	1.004 (0.992–1.016)	0.538
Total protein	131	0.973 (0.936–1.012)	0.168		
Albumin	131	0.904 (0.864–0.946)	**<0.001**	0.921 (0.860–0.987)	**0.019**

Bold values mean *p* < 0.05. KPS, Karnofsky performance status; SCC, squamous cell carcinoma; AC, adenocarcinoma; hCRP, hypersensitive C-reactive protein; EGFR-TKIs, epidermal growth factor receptor-tyrosine kinase inhibitors.

Univariate Cox regression analysis revealed that BMI, sarcopenia, KPS score, hCRP level, and albumin level were significant prognostic factors for PFS (all *p* < 0.05; Table 3). Multivariate analysis confirmed the independent prognostic relevance of BMI (HR, 0.883; 95% CI: 0.814–0.958, *p* = 0.003) and sarcopenia (HR, 2.830; 95% CI: 1.662–4.817, *p* < 0.001) for PFS (Table 3). Univariate and multivariate Cox regression analyses of both the EGFR-TKI and ICI cohorts confirmed that sarcopenia was an independent negative factor for PFS (EGFR-TKI cohort, HR: 2.946, 95% CI: 1.430–6.068, *p* = 0.003, Supplementary Table 5; ICI cohort, HR: 3.567, 95% CI: 1.647–7.724, *p* = 0.001, Supplementary Table 6).

**TABLE 3:** Results of Cox regression analysis for PFS for all patients.

Characteristics	Total (*n*)	Univariate analysis		Multivariate analysis	
		**Hazard ratio (95% CI)**	***p*-value**	**Hazard ratio (95% CI)**	***p*-value**
Sex	131				
Female	54	Reference			
Male	77	1.051 (0.683–1.616)	0.822		
Age	131	0.985 (0.964–1.007)	0.174		
Smoking	131				
No	72	Reference			
Yes	59	0.971 (0.631–1.493)	0.893		
Drinking	131				
No	99	Reference			
Yes	32	0.865 (0.514–1.455)	0.584		
Histopathology	131				
AC	107	Reference			
SCC	24	1.187 (0.689–2.047)	0.537		
Chemotherapy	131				
No	75	Reference			
Yes	56	1.042 (0.681–1.596)	0.849		
EGFR-TKIs therapy	131				
No	54	Reference			
Yes	77	1.372 (0.893–2.110)	0.149		
Body mass index	131	0.908 (0.846–0.974)	**0.007**	0.883 (0.814–0.958)	**0.003**
Sarcopenia status	131				
Non-sarcopenia	96	Reference		Reference	
Sarcopenia	35	3.590 (2.236–5.762)	**<0.001**	2.830 (1.662–4.817)	**< 0.001**
KPS score	131		**<0.001**		
70	10	Reference		Reference	
80	73	0.977 (0.419–2.278)	0.958	1.638 (0.651–4.125)	0.295
90	46	0.281 (0.110–0.714)	**0.008**	0.696 (0.238–2.035)	0.508
100	2	0.000 (0.000–Inf)	0.995	0.000 (0.000–Inf)	0.996
Hemoglobin	131	0.989 (0.978–1.000)	0.055	1.002 (0.987–1.018)	0.773
hCRP	131	1.010 (1.001–1.018)	**0.022**	1.006 (0.994–1.018)	0.347
Total protein	131	0.971 (0.940–1.004)	0.085	0.991 (0.950–1.035)	0.689
Albumin	131	0.930 (0.893–0.968)	**<0.001**	0.977 (0.911–1.047)	0.508

Bold values mean *p* < 0.05. KPS, Karnofsky performance status; SCC, squamous cell carcinoma; AC, adenocarcinoma; EGFR-TKIs, epidermal growth factor receptor-tyrosine kinase inhibitors, hCRP, hypersensitive C-reactive protein.

**FIGURE 3:**
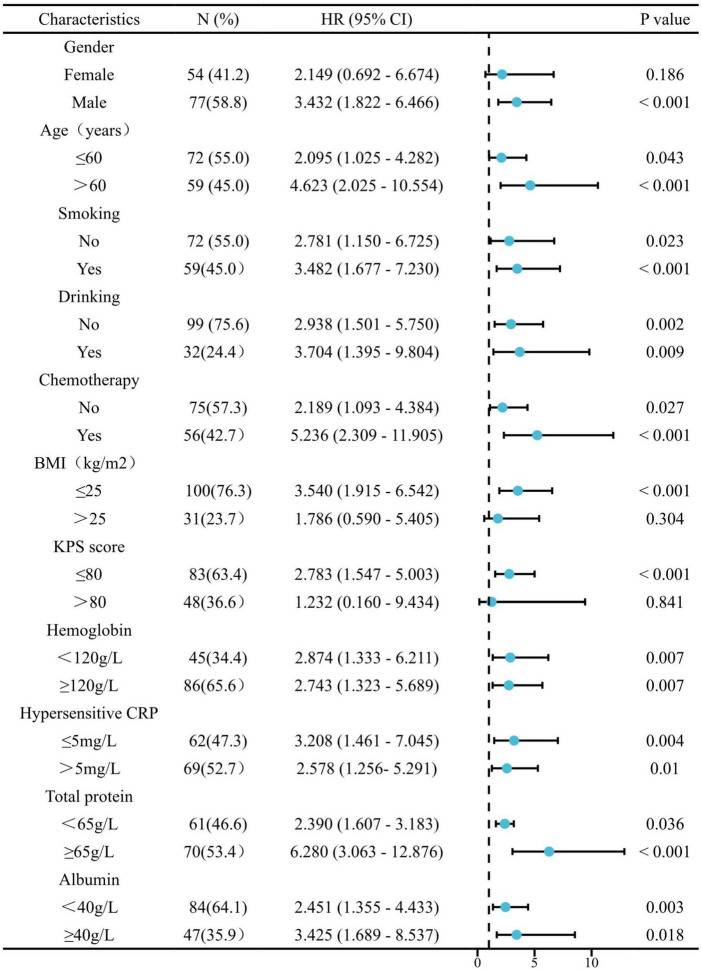
The association between sarcopenia and hazard ratios of OS in various subgroups. BMI, body mass index; KPS, Karnofsky performance status; hCRP, hypersensitive C-reactive protein.

**FIGURE 4:**
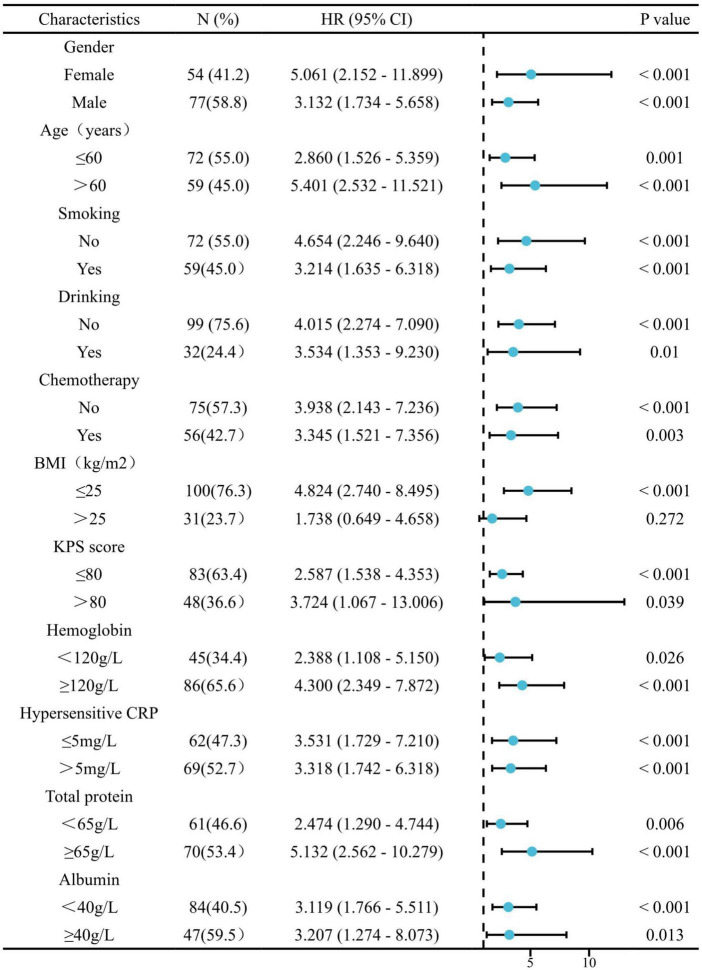
The association between sarcopenia and hazard ratios of PFS in various subgroups. BMI, body mass index; KPS, Karnofsky performance status; hCRP, hypersensitive C-reactive protein.

Stratified analyses were performed to clarify the relationship between sarcopenia and the HRs of OS and PFS in various patient subgroups (Figures 3, 4). Overall, sarcopenia was consistently associated with poor OS and PFS across most subgroups of patients.

### 3.3. OR, DC, and treatment-related AEs

**FIGURE 5:**
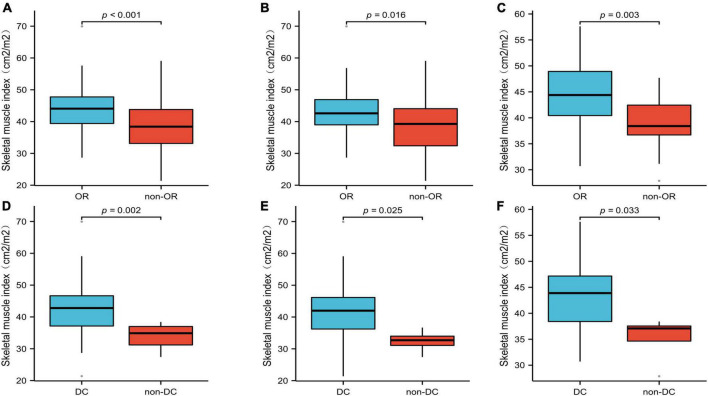
The mean SMI of the OR and DC groups in all patients **(A,D)**, patients treated with EGFR-TKIs **(B,E)**, and patients treated with ICIs **(C,F)**. SMI, skeletal muscle index; OR, objective response; DC, disease control.

Of the 131 patients, 81 had an OR and 125 had DC. The mean SMI was significantly lower in the non-OR group than in the OR group, 38.84 ± 7.11 vs. 43.89 ± 7.55 cm^2^/m^2^, respectively (*p* < 0.001; Figure 5A). Similarly, a significant difference was also found in SMI between the DC group and non-DC group (42.46 ± 7.64 vs. 33.74 ± 4.31 cm^2^/m^2^, *p* = 0.002; Figure 5D). All analyses were repeated in the subgroups of patients treated with EGFR-TKIs or ICIs, and the findings were similar to those of the primary analysis (Figures 5B, C, E, F). The patients in the OR and DC groups had a significantly higher SMI than those in the non-OR and non-DC groups, regardless of whether the patients received EGFR-TKI (Figures 5B, E) or ICI (Figures 5C, F) treatment.

In our study, 51 patients (38.9%) experienced treatment-related AEs: 12 (12/35, 34.3%) in the sarcopenia group and 39 (39/96, 40.6%) in the non-sarcopenia group. There was no statistically significant difference between the two groups (*p* = 0.550). The most frequent AEs were hypothyroidism and skin rashes.

## 4. Discussion

The present study presents considerable real-world data on sarcopenia as a prognostic marker in patients with advanced NSCLC receiving first-line EGFR-TKIs or ICIs. We confirmed that patients with sarcopenia had significantly shorter OS and PFS than those without sarcopenia in the entire patient, EGFR-TKI-treated, and ICI-treated cohorts. In addition, statistically significant differences were observed in mean SMI between the OR and non-OR groups and the DC and non-DC groups. Therefore, nutritional intervention and physical activity programs are recommended to patients with sarcopenia receiving immunotherapy or EGFR-TKI therapy to improve the therapy outcome.

Although there are many studies on the relationship between sarcopenia and immunotherapy in lung cancer, their conclusions are inconsistent. Most of these studies found that a low SMI or a sarcopenia diagnosis is associated with shortened survival in advanced NSCLC patients treated with PD1/PD-L1 checkpoint inhibitors (14, 16, 20–25). However, other studies showed no differences in OS and PFS between patients with and without sarcopenia (26–29). In these studies, ICIs were used in different treatment lines, which may have partially affected the results. The majority of patients included in these studies were treated with second-line or later immunotherapy, whereas only four studies enrolled patients who were receiving first-line immunotherapy, and the sample size in these was relatively small (16, 22, 23, 29). Currently, immunotherapy is increasingly used as the first-line treatment for advanced lung cancer; therefore, our study included advanced lung cancer patients receiving ICIs only as the first-line therapy. Our study included the largest sample size of patients receiving first-line immunotherapy reported to date and provided strong evidence of the negative impact of sarcopenia on the prognosis of lung cancer when using first-line ICIs.

Similarly, using univariate and multivariate analyses of EGFR-TKI subgroups, we found that patients without sarcopenia had significantly longer OS and PFS than those with sarcopenia. In the few studies evaluating the prognostic impact and predictive value of sarcopenia in NSCLC patients harboring EGFR mutations and treated with EGFR-TKIs, as with ICI therapies, the results are inconsistent; however, most studies agree that sarcopenia does not affect PFS and OS (30–32). In a retrospective study conducted by Sabrina et al., sarcopenia did not affect the response to gefitinib in patients with EGFR-mutated NSCLC, even though it was an indicator of poor prognosis in terms of OS (17). In contrast, Atakan et al. found that sarcopenia was an independent factor of poor prognosis for OS and PFS in NSCLC patients receiving EGFR-TKI-targeted therapy (18). These inconsistent results in different studies might come from different study design, different inclusion and exclusion criteria, different sample size and different way of measuring muscle area or definition of sarcopenia, etc.

Next, stratified analyses were performed to clarify the relationship between sarcopenia and the HRs of OS and PFS in various patient subgroups. Overall, sarcopenia was consistently associated with both poor OS and PFS across most subgroups of patients except for patients with BMI > 25kg/m^2^. The reason may be that, for cancer patients, body weight and body fat are also important indicators to reflect the nutritional status of patients and significantly affect the treatment outcome of patients (33). Therefore, for obese (BMI > 25kg/m^2^) cancer patients, a comprehensive body composition analysis may be a better prognostic indicator more than a single myopenia.

The underlying mechanisms by which sarcopenia affects the efficacy of ICIs and EGFR-TKIs are not yet fully understood. Previous studies have found that interleukin-15 is the most abundant cytokine expressed in the skeletal muscle that can regulate CD8^+^ T cells and promote T cell survival (34, 35), which is important for maintaining immune function. Serum interleukin-15 levels decrease in older adults with the loss of muscle mass, suggesting that muscle loss may lead to impaired immune function, which may have some relevance to sarcopenia. Additionally, CD4^+^FoxP3^+^ Tregs infiltrate damaged skeletal muscles, suggesting that sarcopenia may play an important role in tumor immune escape (36). Another possible mechanism for the poor prognosis of NSCLC patients with sarcopenia could be different drug clearance rates in cancer patients with or without sarcopenia, as there is a strong association between pembrolizumab clearance and OS. Patients with high ICI clearance rates had worse survival rates than those with low clearance rates. Some researchers believe the primary method of ICI elimination may be related to the development of cancer cachexia and sarcopenia. Procatabolic status can affect survival by leading to faster protein turnover through monoclonal antibody clearance (37).

The mechanism by which sarcopenia affects the efficacy of EGFR-TKIs is still unclear, but some of the reasons may be similar to those for immunotherapy drugs, such as drug clearance. Retrospective studies have shown that patients with the same body weight and BMI may have different skeletal muscle masses and adipose tissue levels, which could affect EGFR-TKI therapy outcomes (13). When administered, EGFR-TKIs, including gefitinib, are widely distributed in various tissues of the human body, and when bound to human serum albumin and α1-acid glycoprotein, they can have half-lives of up to 48 h. Researchers have demonstrated in animal models that gefitinib is present in lower concentrations in the skin and fat and in higher concentrations in highly perfused organs (38). In addition, studies have shown that gefitinib lasts for up to 96 h in muscle and for only 2 h in fat after oral consumption (39). Therefore, as the diffusion and disposition of drugs in fat are different from those in muscle, this could be one of the mechanisms by which sarcopenia affects the prognosis and toxicity of EGFR-TKIs.

Whether sarcopenia is associated with treatment-related toxicity in lung cancer remains unclear. In this study, we found that the treatment-related toxicities in patients with sarcopenic and non-sarcopenic lung cancer were similar (31, 34). Nie et al. reported that treatment-related toxicity occurred more frequently in patients with sarcopenic lung cancer using afatinib (30). In contrast, Alessio et al. did not find a significant relationship between baseline SMI and AEs (14). The toxicities of EGFR-TKIs or ICIs are closely related to the duration of medication. As the survival times of non-sarcopenia patients were longer than those of sarcopenia patients, this may have affected the incidence of adverse reactions, resulting in the lack of a statistically significant difference between the two groups.

Our study has several strengths. It is the first to include both EGFR-TKIs and ICIs, and the targeted immunotherapy included in our study was a first-line treatment, which conforms to the current standard treatment regimen. In addition, compared with similar studies, ours has the largest number of cases, and there are few studies focusing on both OS and PFS in patients, as in our study.

Our study also has several limitations. First, this was a retrospective, single-center study. Second, sarcopenia was defined only according to SMI and was not based on muscle strength and function, such as grip strength.

## 5. Conclusion

In conclusion, sarcopenia before first-line EGFR-TKI or ICI therapy might be a significant predictor of poor clinical outcomes, leading to shortened OS and PFS and reduced OR and DC. Sarcopenia should be considered before using EGFR-TKIs or ICIs in clinical practice.

## Data availability statement

The original contributions presented in this study are included in the article/Supplementary material, further inquiries can be directed to the corresponding author.

## Ethics statement

The studies involving human participants were reviewed and approved by the Ethics Committee of Sichuan Cancer Hospital. The patients/participants provided their written informed consent to participate in this study.

## Author contributions

JL and TL were responsible for conceptualizing and designing this study, data collection, data interpretation, and manuscript drafting. NY, LX, and XN played a major role in body composition assessment and data analysis. JX, YL, MZ, HZ, CT, SP, LL, HB, CL, and HK participated in acquisition of clinical records, data analysis, and revision of the manuscript. All authors read and approved the final version of manuscript.
