# Supplementary material for: Prognostic value of sarcopenia in patients with lung cancer treated with epidermal growth factor receptor tyrosine kinase inhibitors or immune checkpoint inhibitors
Source: Front Nutr. 2023 Mar 8;10:1113875. doi: 10.3389/fnut.2023.1113875 (PMC10031770; doi:10.3389/fnut.2023.1113875)
Supplement: Supplementary file 1 [file Table_1.DOCX]

Supplementary Table 1 Baseline characteristics of patients receiving EGFR-TKIs

| Characteristics | non-sarcopenia | sarcopenia | P-value |
| --- | --- | --- | --- |
| N | 60（77.9%） | 17（22.1%） |  |
| Gender, n (%) |  |  | 0.102 |
| Female | 38 (63.3%) | 7 (41.2%) |  |
| Male | 22 (36.7%) | 10 (58.8%) |  |
| Smoking, n (%) |  |  | 0.318 |
| No | 45 (75.0%) | 10 (58.8%) |  |
| Yes | 15 (25.0%) | 7 (41.2%) |  |
| Drinking, n (%) |  |  | 0.086 |
| No | 52 (86.7%) | 11 (64.7%) |  |
| Yes | 8 (13.3%) | 6 (35.3%) |  |
| Histopathology, n (%) |  |  | 1.000 |
| AC | 58 (96.7%) | 16 (94.1%) |  |
| SCC | 2 (3.3%) | 1 (5.9%) |  |
| EGFR mutations sites, n (%) |  |  | 0.145 |
| \| Exon 19 \| \| --- \| \| Exon 20 \| \| Exon 21 \| | 34 (56.7%) | 13 (76.5%) |  |
| \| Exon 20 \| \| --- \| \| Exon 20 \| \| Exon 21 \| | 1 (1.7%) | 1 (5.9%) |  |
| \| Exon 21 \| \| --- \| \| Exon 20 \| \| Exon 21 \| | 25 (41.7%) | 3 (17.6%) |  |
| EGFR-TKIs drugs, n (%) |  |  | 0.193 |
| 1^st^ generation | 26 (43.3%) | 5 (29.4%) |  |
| 2^st^ generation | 5 (8.3%) | 0 (0.0%) |  |
| 3^st^ generation | 29 (48.3%) | 12 (70.6%) |  |
| KPS score, n (%) |  |  | **0.016** |
| 70 | 3 (5.0%) | 2 (11.8%) |  |
| 80 | 28 (46.7%) | 12 (70.6%) |  |
| 90 | 29 (48.3%) | 2 (11.8%) |  |
| 100 | 0 (0.0%) | 1 (5.9%) |  |
| Age, median (IQR) | 55.5 (51.75, 65) | 57 (55, 67) | 0.344 |
| BMI, mean ± SD | 22.77 ± 3.29 | 21.849 ± 2.35 | 0.285 |
| Hemoglobin, mean ± SD | 125.78 ± 17.96 | 124.29 ± 17.40 | 0.762 |
| hCRP, median (IQR) | 2.58 (0.67, 7.36) | 7.08 (1.92, 22.04) | 0.142 |
| Total protein, mean ± SD | 66.19 ± 6.09 | 63 ± 5.31 | 0.054 |
| Albumin, mean ± SD | 38.96 ± 4.598 | 36.1 ± 3.37 | **0.019** |

SCC, squamous cell carcinoma; AC, adenocarcinoma; KPS, Karnofsky Performance Status; BMI, body mass index; SD, standard deviation; IQR, interquartile range; hCRP, hypersensitive C-reactive protein.

Supplementary Table 2 Baseline characteristics of patients receiving ICIs

| Characteristic | sarcopenia | non-sarcopenia | P-value |
| --- | --- | --- | --- |
| N | 18（33.3%） | 36（66.7%） |  |
| Gender, n (%) |  |  | 1.000 |
| Female | 3 (16.7%) | 6 (16.7%) |  |
| Male | 15 (83.3%) | 30 (83.3%) |  |
| Smoking, n (%) |  |  | 0.300 |
| No | 4 (22.2%) | 13 (36.1%) |  |
| Yes | 14 (77.8%) | 23 (63.9%) |  |
| Drinking, n (%) |  |  | 0.540 |
| No | 11 (61.1%) | 25 (69.4%) |  |
| Yes | 7 (38.9%) | 11 (30.6%) |  |
| Histopathology, n (%) |  |  | 0.554 |
| AC | 12 (66.7%) | 21 (58.3%) |  |
| SCC | 6 (33.3%) | 15 (41.7%) |  |
| PD-L1 expression, n (%) |  |  | 0.113 |
| ＜1% | 7 (38.9%) | 14 (38.9%) |  |
| 1-50% | 10 (55.6%) | 12 (33.3%) |  |
| >50% | 1 (5.6%) | 10 (27.8%) |  |
| Chemotherapy, n (%) |  |  | 0.298 |
| Yes | 12 (66.7%) | 30 (83.3%) |  |
| No | 6 (33.3%) | 6 (16.7%) |  |
| ICIs drugs, n (%) |  |  | 0.198 |
| PD-1 inhibitors | 15 (83.3%) | 35 (97.2%) |  |
| PD-L1 inhibitors | 3 (16.7%) | 1 (2.8%) |  |
| KPS score, n (%) |  |  | 0.041 |
| 70 | 4 (22.2%) | 1 (2.8%) |  |
| 80 | 12 (66.7%) | 21 (58.3%) |  |
| 90 | 2 (11.1%) | 13 (36.1%) |  |
| 100 | 0 (0.0%) | 1 (2.8%) |  |
| Age, mean ± SD | 58.39 ± 8.06 | 60.33 ± 10.17 | 0.483 |
| Body mass index, median (IQR) | 22.37 ± 3.51 | 23.13 ± 2.58 | 0.421 |
| Hemoglobin, mean ± SD | 121.22 ± 15.22 | 128 ± 18.34 | 0.183 |
| Hypersensitive CRP, median (IQR) | 6.43 (1.34, 27.1) | 5.34(2.18, 17.78) | 0.720 |
| Total protein, mean ± SD | 65.06 ± 3.98 | 64.2 ± 6.55 | 0.552 |
| Albumin, mean ± SD | 36.96 ± 4.49 | 38.11 ± 5.27 | 0.433 |

ICIs, immune checkpoint inhibitors; PD-1, programmed death-1; PD-L1, programmed cell death-ligand 1; KPS, Karnofsky Performance Status; SD, standard deviation; IQR, interquartile range.

Supplementary Table 3. Results of univariable and multivariable analyses of overall survival for patients receiving EGFR-TKIs

| Characteristics | Total(N) | Univariate analysis | |  | Multivariate analysis | |
| --- | --- | --- | --- | --- | --- | --- |
|  |  | Hazard ratio  (95% CI) | P value |  | Hazard ratio  (95% CI) | P value |
| Gender | 77 |  |  |  |  |  |
| Female | 45 | Reference |  |  |  |  |
| Male | 32 | 1.092 (0.559 - 2.133) | 0.796 |  |  |  |
| Age | 77 | 0.975 (0.940-1.011) | 0.176 |  |  |  |
| Smoking | 77 |  |  |  |  |  |
| No | 55 | Reference |  |  |  |  |
| Yes | 22 | 0.938 (0.449-1.958) | 0.864 |  |  |  |
| Drinking | 77 |  |  |  |  |  |
| No | 63 | Reference |  |  |  |  |
| Yes | 14 | 1.100 (0.478 - 2.530) | 0.823 |  |  |  |
| Histopathology | 77 |  |  |  |  |  |
| AC | 74 | Reference |  |  |  |  |
| SCC | 3 | 0.827 (0.113 - 6.063) | 0.852 |  |  |  |
| Chemotherapy | 77 |  |  |  |  |  |
| No | 63 | Reference |  |  |  |  |
| Yes | 14 | 0.740 (0.307 - 1.786) | 0.503 |  |  |  |
| BMI | 77 | 0.914 (0.814 - 1.025) | 0.125 |  |  |  |
| Sarcopenia status | 77 |  |  |  |  |  |
| non-sarcopenia | 60 | Reference |  |  | Reference |  |
| sarcopenia | 17 | 3.364 (1.576 - 7.178) | **0.002** |  | 2.806 (1.304 - 6.037) | **0.008** |
| KPS score | 77 |  |  |  |  |  |
| 70 | 5 | Reference |  |  |  |  |
| 80 | 40 | 1.160 (0.269 - 4.998) | 0.842 |  |  |  |
| 90 | 31 | 0.592 (0.132 - 2.646) | 0.492 |  |  |  |
| 100 | 1 | 0.000 (0.000 - Inf) | 0.997 |  |  |  |
| Hemoglobin | 77 | 0.989 (0.971-1.006) | 0.203 |  |  |  |
| hCRP | 77 | 1.005 (0.992-1.019) | 0.441 |  |  |  |
| Total protein | 77 | 0.968 (0.920-1.018) | 0.203 |  |  |  |
| Albumin | 77 | 0.904 (0.851 - 0.961) | **0.001** |  | 0.910 (0.853 - 0.972) | **0.005** |

SCC, squamous cell carcinoma; AC, adenocarcinoma; KPS, Karnofsky Performance Status; BMI, body mass index; hCRP, hypersensitive C-reactive protein.

Supplementary Table 4. Results of univariable and multivariable analyses of overall survival for patients receiving ICIs

| Characteristics | Total(N) | Univariate analysis | |  | Multivariate analysis | |
| --- | --- | --- | --- | --- | --- | --- |
|  |  | Hazard ratio  (95% CI) | P value |  | Hazard ratio  (95% CI) | P value |
| Gender | 54 |  |  |  |  |  |
| Female | 9 | Reference |  |  |  |  |
| Male | 45 | 1.039 (0.396 - 2.727) | 0.938 |  |  |  |
| Age | 54 | 1.043 (1.002 - 1.087) | **0.042** |  | 1.031 (0.980 - 1.084) | 0.242 |
| Smoking | 54 |  |  |  |  |  |
| No | 17 | Reference |  |  |  |  |
| Yes | 37 | 1.055 (0.479-2.324) | 0.895 |  |  |  |
| Drinking | 54 |  |  |  |  |  |
| No | 36 | Reference |  |  |  |  |
| Yes | 18 | 1.133 (0.522-2.460) | 0.752 |  |  |  |
| Histopathology | 54 |  |  |  |  |  |
| SCC | 21 | Reference |  |  |  |  |
| AC | 33 | 0.974 (0.456-2.081) | 0.946 |  |  |  |
| Chemotherapy | 54 |  |  |  |  |  |
| No | 12 | Reference |  |  |  |  |
| Yes | 42 | 0.924 (0.393-2.173) | 0.856 |  |  |  |
| BMI | 54 | 0.913 (0.801-1.039) | 0.169 |  |  |  |
| sarcopenia status | 54 |  |  |  |  |  |
| non-sarcopenia | 36 | Reference |  |  | Reference |  |
| sarcopenia | 18 | 3.006 (1.487-6.077) | **0.002** |  | 2.155(1.107 - 4.484) | **0.028** |
| KPS score | 54 | 0.818 (0.745-0.899) | **<0.001** |  | 0.850 (0.769-0.939) | **0.001** |
| 70 | 5 | Reference |  |  | Reference |  |
| 80 | 33 | 0.058 (0.016 - 0.212) | **< 0.001** |  | 0.069 (0.015 - 0.330) | **< 0.001** |
| 90 | 15 | 0.016 (0.003 - 0.081) | **< 0.001** |  | 0.025 (0.004 - 0.167) | **< 0.001** |
| 100 | 1 | 0.000 (0.000 - Inf) | 0.997 |  | 0.000 (0.000 - Inf) | 0.997 |
| Hemoglobin | 54 | 0.970 (0.950-0.991) | **0.005** |  | 0.979 (0.949-1.010) | 0.188 |
| hCRP | 54 | 1.018 (1.002 - 1.035) | **0.025** |  | 1.003 (0.983 - 1.023) | 0.760 |
| Total protein | 54 | 0.987 (0.927 - 1.050) | 0.676 |  |  |  |
| Albumin | 54 | 0.917 (0.858 - 0.981) | **0.011** |  | 1.006 (0.890 - 1.137) | 0.921 |

SCC, squamous cell carcinoma; AC, adenocarcinoma; KPS, Karnofsky Performance Status; BMI, body mass index; hCRP, hypersensitive C-reactive protein.

Supplementary Table 5. Results of univariable and multivariable analyses of progression free survival for patients receiving EGFR-TKIs

| Characteristics | Total(N) | Univariate analysis | |  | Multivariate analysis | |
| --- | --- | --- | --- | --- | --- | --- |
|  |  | Hazard ratio  (95% CI) | P value |  | Hazard ratio  (95% CI) | P value |
| Gender | 77 |  |  |  |  |  |
| Female | 45 | Reference |  |  |  |  |
| Male | 32 | 1.137 (0.642 - 2.011) | 0.660 |  |  |  |
| Age | 77 | 0.974 (0.944 - 1.005) | 0.095 |  | 0.981 (0.948 - 1.016) | 0.293 |
| Smoking | 77 |  |  |  |  |  |
| No | 55 | Reference |  |  |  |  |
| Yes | 22 | 1.035 (0.555 - 1.930) | 0.914 |  |  |  |
| Drinking | 77 |  |  |  |  |  |
| No | 63 | Reference |  |  |  |  |
| Yes | 14 | 0.753 (0.337 - 1.682) | 0.489 |  |  |  |
| Histopathology | 77 |  |  |  |  |  |
| AC | 74 | Reference |  |  |  |  |
| SCC | 3 | 1.114 (0.270 - 4.600) | 0.882 |  |  |  |
| Chemotherapy | 77 |  |  |  |  |  |
| No | 63 | Reference |  |  |  |  |
| Yes | 14 | 0.837 (0.406 - 1.729) | 0.631 |  |  |  |
| BMI | 77 | 0.906 (0.829 - 0.990) | **0.029** |  | 0.890 (0.801 - 0.988) | **0.029** |
| sarcopenia status | 77 |  |  |  |  |  |
| non-sarcopenia | 60 | Reference |  |  | Reference |  |
| sarcopenia | 17 | 3.368 (1.768 - 6.415) | **< 0.001** |  | 2.946 (1.430 - 6.068) | **0.003** |
| KPS score |  |  |  |  |  |  |
| 70 | 5 | Reference |  |  | Reference |  |
| 80 | 40 | 0.933 (0.326 - 2.671) | 0.897 |  | 1.466 (0.444 - 4.843) | 0.530 |
| 90 | 31 | 0.335 (0.108 - 1.043) | 0.059 |  | 0.787 (0.208 - 2.975) | 0.725 |
| 100 | 1 | 0.000 (0.000 - Inf) | 0.997 |  | 0.000 (0.000 - Inf) | 0.997 |
| Hemoglobin | 77 | 0.990 (0.976 - 1.004) | 0.151 |  |  |  |
| hCRP | 77 | 1.011 (1.001 - 1.022) | **0.026** |  | 1.004 (0.991 - 1.018) | 0.539 |
| Total protein | 77 | 0.952 (0.911 - 0.994) | **0.027** |  | 1.007 (0.952 - 1.064) | 0.812 |
| Albumin | 77 | 0.918 (0.872 - 0.966) | **0.001** |  | 0.956 (0.886 - 1.032) | 0.252 |

SCC, squamous cell carcinoma; AC, adenocarcinoma; KPS, Karnofsky Performance Status; BMI, body mass index; hCRP, hypersensitive C-reactive protein.

Supplementary Table 6. Results of univariable and multivariable analyses of progression free survival for patients receiving ICIs

| Characteristics | Total(N) | Univariate analysis | |  | Multivariate analysis | |
| --- | --- | --- | --- | --- | --- | --- |
|  |  | Hazard ratio  (95% CI) | P value |  | Hazard ratio  (95% CI) | P value |
| Gender | 54 |  |  |  |  |  |
| Female | 9 | Reference |  |  |  |  |
| Male | 45 | 0.585 (0.274 - 1.251) | 0.167 |  |  |  |
| Age | 54 | 0.999 (0.967 - 1.031) | 0.934 |  |  |  |
| Smoking | 54 |  |  |  |  |  |
| No | 17 | Reference |  |  |  |  |
| Yes | 37 | 0.661 (0.343 - 1.277) | 0.218 |  |  |  |
| Drinking | 54 |  |  |  |  |  |
| No | 36 | Reference |  |  |  |  |
| Yes | 18 | 0.842 (0.413 - 1.718) | 0.637 |  |  |  |
| Histopathology | 54 |  |  |  |  |  |
| AC | 21 | Reference |  |  |  |  |
| SCC | 33 | 0.957 (0.492 - 1.861) | 0.896 |  |  |  |
| Chemotherapy | 54 |  |  |  |  |  |
| No | 12 | Reference |  |  |  |  |
| Yes | 42 | 1.272 (0.578 - 2.800) | 0.550 |  |  |  |
| BMI | 54 | 0.895 (0.794 - 1.009) | 0.070 |  | 0.897 (0.794 - 1.012) | 0.078 |
| Sarcopenia statuss | 54 |  |  |  |  |  |
| non-sarcopenia | 36 | Reference |  |  | Reference |  |
| sarcopenia | 18 | 3.667 (1.788 - 7.518) | **< 0.001** |  | 3.567 (1.647 - 7.724) | **0.001** |
| KPS score | 54 |  | **< 0.001** |  |  |  |
| 70 | 5 | Reference |  |  | Reference |  |
| 80 | 33 | 0.789 (0.184 - 3.380) | 0.750 |  | 1.035 (0.230 - 4.665) | 0.964 |
| 90 | 15 | 0.181 (0.035 - 0.934) | **0.041** |  | 0.281 (0.050 - 1.579) | 0.149 |
| 100 | 1 | 0.000 (0.000 - Inf) | 0.997 |  | 0.000 (0.000 - Inf) | 0.997 |
| Hemoglobin | 54 | 0.987 (0.968 - 1.006) | 0.164 |  |  |  |
| hCRP | 54 | 1.007 (0.991 - 1.023) | 0.392 |  |  |  |
| Total protein | 54 | 1.008 (0.955 - 1.064) | 0.772 |  |  |  |
| Albumin | 54 | 0.954 (0.897 - 1.016) | 0.142 |  |  |  |

SCC, squamous cell carcinoma; AC, adenocarcinoma; KPS, Karnofsky Performance Status; BMI, body mass index; hCRP, hypersensitive C-reactive protein.
