# Supplementary material for: Prognostic value of sarcopenia in patients with lung cancer treated with epidermal growth factor receptor tyrosine kinase inhibitors or immune checkpoint inhibitors
Source: Front Nutr. 2023 Mar 8;10:1113875. doi: 10.3389/fnut.2023.1113875 (PMC10031770; doi:10.3389/fnut.2023.1113875)
Supplement: Supplementary file 2 [file Image_1.pdf]

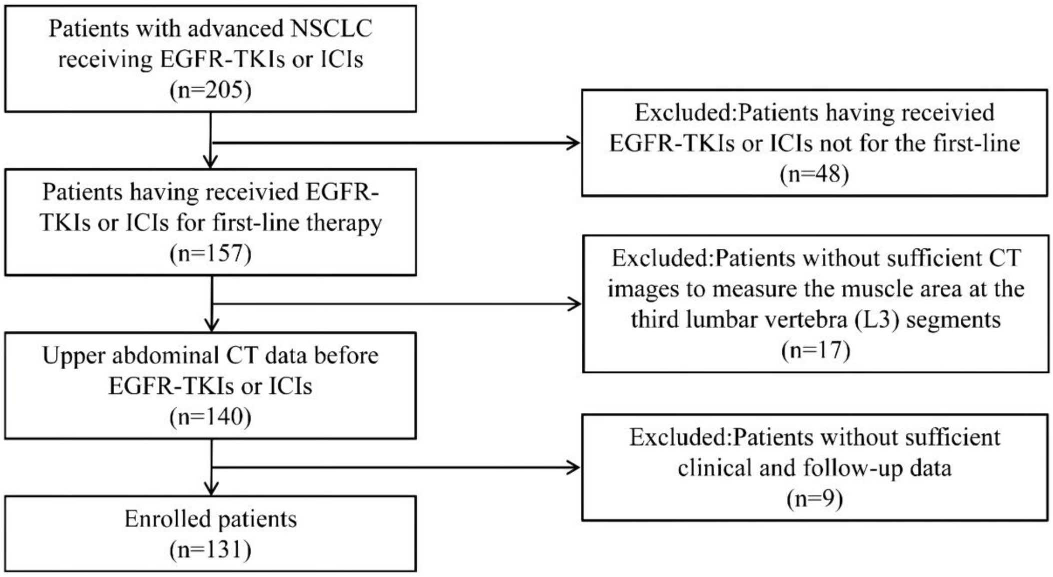

Supplementary Figure 1. CONSORT flow diagram showing the process for enrolment and the number of patients involved at each inclusion/exclusion stage.
