# Supplementary material for: Prognostic value of sarcopenia in patients with lung cancer treated with epidermal growth factor receptor tyrosine kinase inhibitors or immune checkpoint inhibitors
Source: Front Nutr. 2023 Mar 8;10:1113875. doi: 10.3389/fnut.2023.1113875 (PMC10031770; doi:10.3389/fnut.2023.1113875)
Supplement: Supplementary file 3 [file Image_2.pdf]

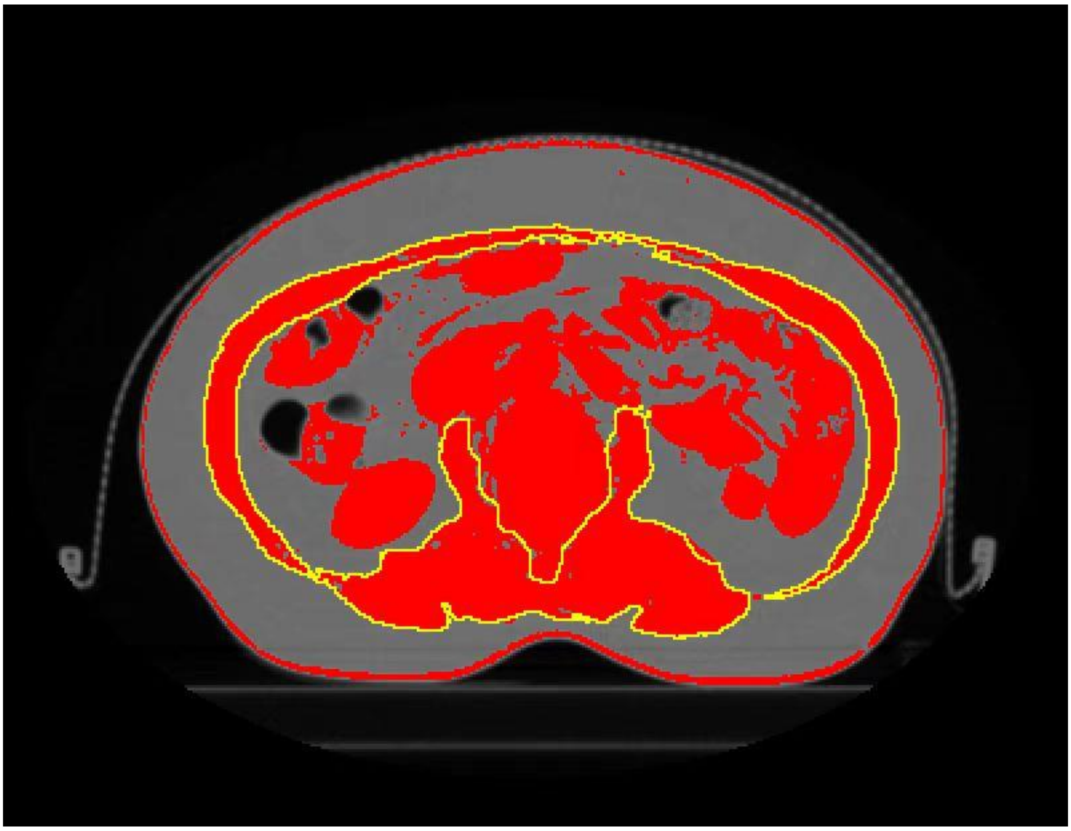

Supplementary Figure 2. Skeletal muscle mass (enclosed by the yellow line) analysis of computed tomography images on an L3 section by SliceOmatic.
